# Evaluating the Influence of Different Artificial Diets on *Apis mellifera* L. Using Health Biomarkers and Performance Metrics

**DOI:** 10.3390/insects15110905

**Published:** 2024-11-19

**Authors:** Shams Ul Islam, Muhammad Anjum Aqueel, Muhammad Usman Yousuf, Asim Abbasi, Muhammad Yasin, Rashid Iqbal, Muhammad Fahim Raza, Aqsa Parvaiz, Nazih Y. Rebouh

**Affiliations:** 1 Department of Entomology, Faculty of Agriculture and Environment, The Islamia University of Bahawalpur, Bahawalpur 63100, Pakistan; 2 Division of Biology, Silwood Park Campus, Imperial College London, Ascot SL5 7PY, UK; 3 Department of Entomology, University of Agriculture, Faisalabad 38040, Pakistan; asimuaf95@gmail.com; 4 Chinese Academy of Tropical Agricultural Sciences, Coconut Research Institute, Wenchang 571339, China; 5 Department of Agronomy, Faculty of Agriculture and Environment, The Islamia University of Bahawalpur, Bahawalpur 63100, Pakistan; 6 Department of Life Sciences, Western Caspian University, Baku AZ1001, Azerbaijan; 7 Department of Veterinary Pathology, Western College of Veterinary Medicine, University of Saskatchewan, Saskatoon, SK S7N 5B4, Canada; 8 Department of Biochemistry and Biotechnology, The Women University Multan, Multan 66000, Pakistan; 9 Department of Environmental Management, Institute of Environmental Engineering, RUDN University, 6 Miklukho-Maklaya St., Moscow 117198, Russia

**Keywords:** *Apis mellifera* diet, artificial diets, foraging activity, honey quality, social interactions of *Apis mellifera*, health biomarkers

## Abstract

The diet of *Apis mellifera* L. is a crucial factor for managing its colonies particularly during dearth periods. Numerous diets have been developed; however, their global implementation faces challenges due to diverse climatic conditions and some other factors. To address this issue, three previously evaluated diets (selected from seven) were tested to assess their effects on *A. mellifera* using key health biomarkers (immune function, stress response) and performance metrics (foraging activity, honey quality, and social interactions). The experiment was conducted using 12 colonies, including three replications, in The Islamia University of Bahawalpur, Pakistan, from June to September 2023. The results revealed that all the tested parameters were significantly affected by diets. Highest phenol-oxidase activity was recorded in T1 (28.7 U/mg). Heat shock protein (HSP) bands showed that T1 had the fewest (Hsp70), while T0 had more bands (Hsp40, Hsp60, and Hsp70), indicating stress differences. In foraging activity, average number of outgoing bees were highest in T1 (81.8) and lowest in T0 (31.2) and similar trend was followed for returning bees, i.e., T1 (81.8) and T0 (31.2). For pollen-carrying bees, the highest bees were counted in T1 (34.9), and the lowest in T0 (4.10). Honey quality was also significantly affected by diets, pH was highest in T1 (3.85), while moisture was highest in T0 (19.44%). Diastase activity, ash content, and electrical conductivity were best in T1 (13.74 units/g, 0.17%, 0.94 mS/cm, respectively). Mineral content was highest in T1 (406.54 mg/kg), and fructose content also peaked in T1 (396.21 mg/kg). Antioxidant contents, total phenolic content, flavonoid content, and ORAC value were highest in T1 (60.50 mg GAE/100 g, 44.41 mg QE/100 g, 10,237.30 µmol TE/g), while T0 consistently showed the lowest values across all parameters. In social interaction experiments, trophallaxis events were most frequent in T1 (7.38), and T1 also exhibited the longest trophallaxis time (5.51 s). The number of bees per trophallaxis event and antennation frequency followed a similar trend, with the highest recorded in T1 (5.16 bees/event, 10.1 antennation frequency) and the lowest in T0 (2.94 bees/event, 4.18 antennation frequency). Therefore, diet-1 (Watermelon juice 20 mL + Fenugreek powder 2 g + Chickpea flour 20 g + Lupin flour 20 g + Mung bean flour 20 g + Yeast 10 g + Powdered sugar 40 g + vegetable oil 10 mL) is recommended as a suitable substitute for managing *A. mellifera* colonies during dearth periods.

## Simple Summary

Effects of artificial diets on *Apis mellifera* L. using health biomarkers and performance metrics, during a dearth period, focusing on immune function, stress response, foraging activity, honey quality, and social interactions were evaluated in this study. The experiments comprised four diets (three artificial diets and one control diet) to be tested on 12 colonies with three replications. As a result, significant differences were found among all the treatments, with T1 (diet-1: Watermelon Juice 20 mL + Fenugreek powder 2 g + Chickpea flour 20 g + Lupin flour 20 g + Mung bean flour 20 g + Yeast 10 g + powdered Sugar 40 g + vegetable Oil 10 mL) consistently performing the best in all the parameters. It exhibited the highest phenol-oxidase activity, lowest heat shock protein levels, and higher foraging activity (outgoing, returning bees and carrying more pollen). Bees on T1 produced higher quality honey, showing the best pH, diastase activity, ash content, mineral content, and antioxidant properties. Social interactions, including trophallaxis events, trophallaxis time, gathering in each trophallaxis event and antennation frequency, were also most frequent in T1. Conversely, T0 showed the poorest results across all parameters. Based on the findings, T1 is recommended for its positive impact on *Apis mellifera* using both health biomarkers and colony performance metrics, making it a suitable substitute diet for sustaining honeybee colonies during periods of nectar and pollen deficiency.

## 1. Introduction

*Apis mellifera* L. is famed for its honey production and pollination services for a variety of crops worldwide [1]. Beyond this, beeswax, royal jelly, and propolis produced by *A. mellifera*, offer significant benefits to humans due to their medicinal properties [2]. Beekeepers endure substantial colony losses due to different stresses associated with pathogens and pesticides during long-distance transportation particularly in periods of food scarcity [3]. However, food scarcity is a primary factor that leads to impaired immune function and increased susceptibility to disease and pests [4] and adversely affects brood production and colony survival [5,6].

Nectar is a source of carbohydrates [5] and provides energy to honeybees for their daily activities (i.e., foraging or hive maintenance). Honeybees convert nectar into honey through a process involving digestion, enzymatic activity, and evaporation [7]. Pollen is the primary source of proteins, lipids, and other nutrients [5] and is a primary concern due to its critical role in brood development, colony growth and resilience, and disease and pest resistance [8].

In periods of food scarcity, beekeepers often provide their colonies with artificial diets to support colony development and maintain health [9,10,11,12]. Some beekeepers also provide fruit supplements and sugar syrup to sustain colony development and brood growth [13,14]. The protein content should be the main focus during artificial diet development due to its importance in maintaining brood health [15]. Many artificial diets were formulated in previous studies, using various protein sources, such as casein, fish meal, egg yolk powder, pea powder, soybean flour, dried gram, brewer’s yeast, guar meal, and skim milk powder [16,17].

Honeybees prefer to feed on artificial diets presented in the form of a candy-like dough patty [18,19]. These prepared artificial diets were placed inside the hive, allowing easy access for the bees to feed. Protein-enriched diets enable bees to groom and develop their broods [17]. Colony health biomarkers and performance metrics are important parameters for evaluating diet efficacy [3,20]. Scientists select honeybee samples from colonies fed artificial diets to assess the quality of the nutritional resources provided to the colonies [3]. The performance of a honeybee colony can be estimated by evaluating its foraging activity [20], social interactions [21] and the quality of the honey produced [22]. Health biomarkers such as phenol-oxidase (PO) activity and heat shock proteins have been used to assess bee immune function and stress response, respectively [23,24]. Phenol-oxidase activity is a key component of immune responses in the hemolymph of invertebrates [24] and is directly related to the type of diet honeybees are fed; optimal diet composition means increased PO activity [25]. Phenol-oxidase (PO) activity plays a vital role in the overall immune system of insects, including honeybees, as it is involved in defense mechanisms within the hemolymph [26,27,28,29].

The aim of this study was to compare the effects of different artificial diets on *A. mellifera* colonies in Bahawalpur, Pakistan, during the period of food deficiency. The colonies were subjected to various feeding treatments, with three replications. The performance metrics, including foraging activity, social interactions (such as antennation and trophallaxis), honey quality, and health biomarkers (immune function, stress response, and metabolic health) were monitored during the course of the study.

We hypothesized that colonies fed on artificial diets would exhibit better performance in foraging activity, honey quality and social interactions (trophallaxis and antennation), and also will be prominent in health biomarkers in both immune function and stress response as compared to the control treatment.

## 2. Materials and Methods

### 2.1. Colony Setup and Management

The study was conducted in the apiculture field of the Department of Entomology at The Islamia University of Bahawalpur. The experiments were carried out from June to September 2023, a period known for the dearth of natural forage. Colony setup consisted of 12 single-story colonies of *A. mellifera* species. Colonies were divided into four treatment groups with three replications. All the colonies had 9 frames with a fertile queen and approximately 20,000 bee population. Three different artificial diets (T1 (diet-1): Watermelon Juice 20 mL + Fenugreek Powder 2 g + Chickpea Flour 20 g + Lupin Flour 20 g + Mung Bean Flour 20 g + Yeast 10 g + Powdered Sugar 40 g + Vegetable Oil 10 mL; T2 (diet-2): Watermelon Juice 20 mL + Fenugreek Powder 2 g + Chickpea Flour 30 g + Mung Bean Flour 30 g + Yeast 10 g + Powdered Sugar 40 g + Vegetable Oil 10 mL; T3 (diet-3): Watermelon Juice 20 mL + Fenugreek Powder 2 g + Chickpea Flour 30 g + Lupin Flour 30 g + Yeast 10 g + Powdered Sugar 40 g + Vegetable Oil 10 mL) were prepared in a form of candy-like dough, and provided to the colonies for feeding along with one control treatment T0 (1 L of 50% sugar solution). Artificial diets were provided to the colonies for eight weeks, with all diets replaced weekly with fresh preparations. During eight weeks of feeding and after eight weeks of feeding, social interactions, colony foraging activity and honey quality were measured. In August 2023, colonies were sampled for biochemical assays.

### 2.2. Immune Function

To measure PO activity, 15 isolated nurse honeybees were collected from the middle frames of the hive, from each treatment (3 from each replication). Thoraxes of these collected bees were separated for analysis [13]. A 20 µL of thorax supernatant was taken and placed into a cuvette. Then, 505 µL of phosphate saline buffer (pH 7.4) and 675 µL of milli-Q^®^ water was added to this [4]. The mixture was incubated at 37 °C for 5 min. After incubation, 300 µL of L-DOPA solution (2 mg/mL) was added, and absorbance was measured at 490 nm at 0 and 10 min. PO activity was expressed as milliunits per milligram of tissue (mU/mg) [25].

### 2.3. Stress Response

For this experiment, 15 forager honeybees were collected from each treatment (3 from each replication) at the entrance of the colony. Temperature during the time of collection was between 32 °C and 36 °C. Bees from each diet group were kept at 45 °C for 4 h in an incubator, as this period was chosen to induce maximum heat shock protein (HSP) expression [30]. After the heat stress treatment, 20 µL of hemolymph was extracted (by puncturing the thorax and pressing gently on abdomen) and collected (with the help of micropipette) from each bee. The extracted hemolymph was stored in a buffer solution (Tris-HCl 50 mM, 10% SDS, 50% glycerol, distilled water, and 2-mercaptoethanol) at −20 °C for further analysis. Hemolymph samples from each group were subjected to SDS-PAGE (Sodium Dodecyl Sulfate Polyacrylamide Gel Electrophoresis), to assess the expression of HSPs. In total, 10 µL volume of each hemolymph sample was loaded on 8% acrylamide gel. Electrophoresis was performed with a constant current of 20 mA for 5 h to separate proteins by molecular weight [31]. Protein molecular weight markers were also loaded onto the gel to assess the size of the exposed protein’s molecules [29]. After electrophoresis, the gel was stained with Coomassie blue solution and photographed using a gel documentation system [30]. The protein bands were analyzed to compare the expression of HSPs from the different diet groups.

### 2.4. Foraging Activity

In this experiment, foraging activity (number of outgoing bees, returning bees to their hives and containing pollens while returning) was determined by visually observing the hives twice a week to count the number of bees leaving the hive [32], returning to the hive [33], and carrying pollen [29]. To record the data, observations were conducted for 5 min each [34], between 8:00 a.m. and 10:00 a.m., when foraging activity is at its peak [35].

### 2.5. Honey Quality

At the end of the experiments, honey was extracted and 250 g from all the treatments and control group were separated for analysis. Honey samples were kept at room temperature for pH, moisture and sugar content analysis, while for enzymatic analysis the samples were kept at 4 °C. The pH was measured by dissolving the honey in the distilled water and by using pH meter (Dr. Meter pH-100), moisture content, diastase activity and ash content of honey were measured using the methods described by Bogdanov et al. [36]. Electrical conductivity, fructose content, phenol content, mineral content, and oxygen radical activity capacity (ORAC) were measured using the standard methods as described by Aazza et al. [37]. Flavonoid contents were measured using the method similar to Popova et al. [38].

### 2.6. Social Interactions

In social interactions, the activities of honeybee colonies were observed during the 8-week period of feeding artificial diets. Trophallaxis and antennation behaviors between worker honeybees were recorded using a high-definition camera Go-Pro Hero (CHDRB-101-CN) on a selected frame in the hive. The camera was set up at a fixed position to capture detailed interactions, ensuring minimal disturbance to the colonies. An appropriate light source was used to ensure clear visibility without disrupting the natural behavior of the bees. A low-intensity, non-intrusive light was employed to illuminate the hive sufficiently for the camera to capture high-quality footage. The position of the light was kept cleverly to avoid direct exposure to the bees to minimize stress and behavioral responses of bees [39]. The recorded footage and videos were analyzed to assess trophallaxis events, trophallaxis duration, the number of bees involved in each event and antennation frequency [39], the results were compared to control treatments for statistical analysis.

### 2.7. Statistical Analysis

All statistical analyses and graphical representations were conducted using R (version 4.4.0; R Core Team, 2024), utilizing the ggplot2 package for data visualization. A Completely Randomized Design (CRD) was employed alongside Tukey’s HSD test to assess PO activity at a significance level of 0.05. Statistical analysis of foraging activity was performed using a two-way ANOVA, followed by the Least Significant Difference (LSD) test, at 5% significance level (*p* < 0.05). The quality parameters of honey were evaluated using CRD with the LSD test for post hoc comparisons. Additionally, a two-way factorial design was applied to analyze trophallaxis and antennation behaviors, with Tukey’s HSD test used for post hoc analyses at a 0.05 significance level. To assess the relationship between trophallaxis and antennation behavior, Pearson’s correlation matrix was calculated using same software R (version 4.4.0; R Core Team, 2024).

## 3. Results

### 3.1. Immune Function (Phenol-Oxidase Activity)

**Figure 1:**
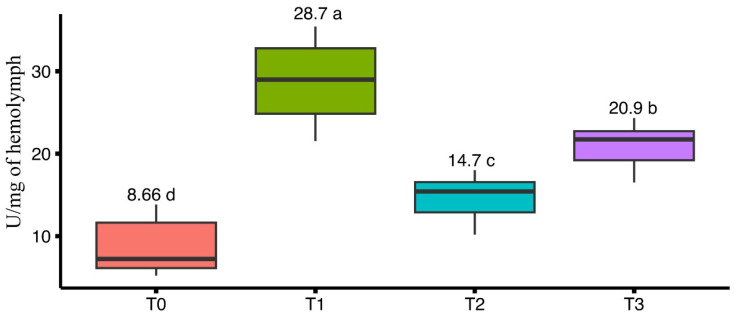
Effect of different artificial diets on phenol-oxidase activity to measure the immune functions. Bars having different lower-case letters are significantly different at probability level of 5%.

The impact of four different artificial diets on phenol-oxidase activity was measured, after feeding the bees with artificial diets for a period of 8 weeks. A statistically significant difference was observed in the average PO activity (U/mg of hemolymph) among the treatments (F_(3, 56)_ = 99.86, *p* < 0.001). Honeybees from treatment T1 exhibited the highest average PO activity (28.7 ± 1.20 U/mg of hemolymph), followed by T3 (20.9 ± 0.65 U/mg of hemolymph) and T2 (14.7 ± 0.66 U/mg of hemolymph). The lowest average PO activity was observed in T0 (8.66 ± 0.81 U/mg of hemolymph) (Figure 1).

### 3.2. Stress Responses (Heat Shock Proteins)

**Figure 2:**
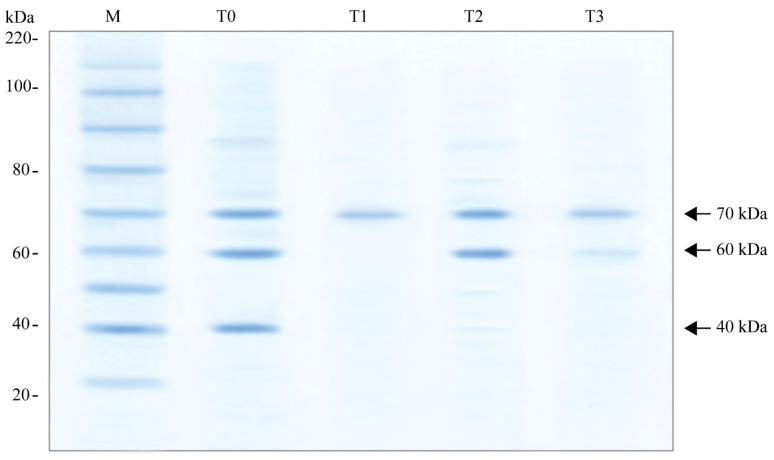
SDS-PAGE analysis of heat shock-induced proteins (HSPs) from the hemolymph of forager bees. The gel was stained using Coomassie blue dye. ‘M’ represents the molecular weight marker (kDa). Identified HSPs are marked with arrows on the right side of the gel.

SDS-PAGE was conducted on samples from all diets, after exposing bees to heat stress at 45 °C (Table 1). The gel was stained with Coomassie Blue to visualize the HSP bands. The results showed that T0 exhibited the highest number of HSP bands (HSPs40, HSPs60 and HSPs70), indicating the greatest stress level. T1 showed the fewest bands (HSPs70), reflecting the lowest stress. The T2 and T3 groups displayed intermediate band counts (HSPs60 and HSPs70), with T2 having slightly more bands than T3 (HSPs60 and HSPs70) but less than T0 (Figure 2).

### 3.3. Foraging Activity

The impact of four different artificial diets on the number of foraging bees over an eight-week period was assessed in terms of honeybees going out of the hive for foraging. All treatments showed a significant difference (F_(3, 158)_ = 771.19, *p* < 0.001). Treatment T1 exhibited the highest average number of outgoing bees (81.8 ± 1.37) over the eight weeks, followed by T3 (61.2 ± 1.28) and T2 (50.9 ± 1.06), with the lowest average number of outgoing bees observed in T0 (31.2 ± 0.664).

**Figure 3:**
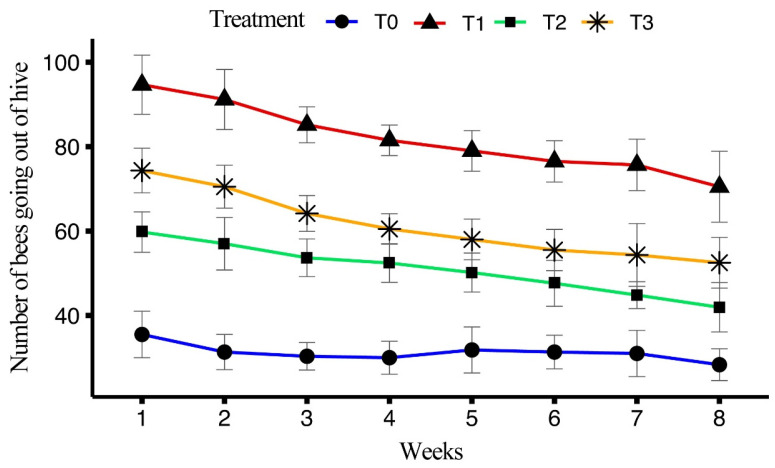
Effect of different artificial diets on foraging activity in terms of outgoing bees over the period of eight weeks.

In T1, the maximum number of outgoing bees was recorded in week 1 (94.67 ± 2.86), followed by weeks 2 and 3, while the minimum number of outgoing bees was observed in week 8 (70.5 ± 3.45). In T2, the highest number of outgoing bees was recorded in week 1 (59.76 ± 1.96), with weeks 2 and 3 following and the lowest number was seen in week 8 (41.94 ± 2.39). In T3, the maximum number of outgoing bees was observed in week 1 (74.33 ± 2.16), with weeks 2 and 3 also showing high numbers, and the minimum was recorded in week 8 (52.50 ± 2.46). For T0 (control group), the highest number of outgoing bees was recorded in week 1 (35.50 ± 2.25), with weeks 5 and 6 following and the lowest was observed in week 8 (28.33 ± 1.54). The average number of outgoing bees over the eight weeks is shown in Figure 3.

All treatments showed a significant difference (F_(3, 158)_ = 639.23, *p* < 0.001) in terms of honeybees returning back to the hive. Treatment T1 had the highest average number of returning bees (81.8 ± 1.37) over the eight weeks, followed by T3 (61.2 ± 1.28) and T2 (50.9 ± 1.06). The lowest average number of returning bees was observed in T0 (31.2 ± 0.66).

**Figure 4:**
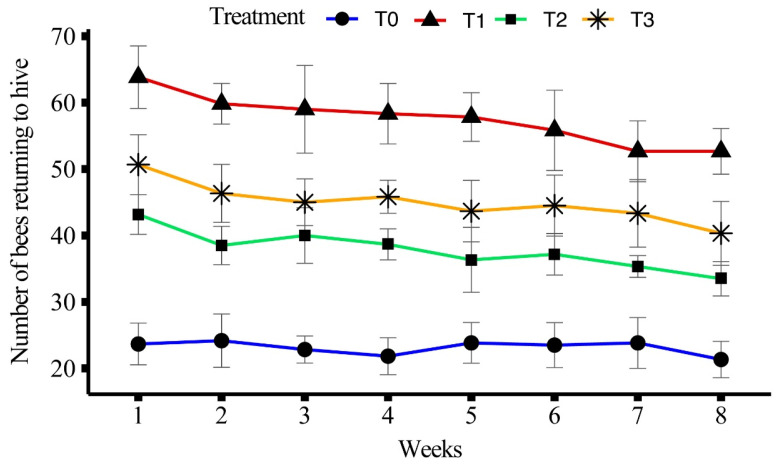
Effect of different artificial diets on foraging activity in terms of honeybees returning back to hive over a period of eight weeks.

In T1, the maximum number of returning bees was recorded in week 1 (63.83 ± 1.92), followed by weeks 2 and 3, while the minimum was observed in week 8 (52.67 ± 1.41). For T2, the highest number of returning bees was recorded in week 1 (43.17 ± 1.22), with a gradual decline over the weeks, and the lowest was in week 8 (33.50 ± 1.06). In T3, the maximum number of returning bees was recorded in week 1 (50.67 ± 1.84) and the lowest was recorded in week 8 (40.33 ± 1.96). T0 recorded the highest number of returning bees in week 5 (23.83 ± 1.25), with the lowest recorded in week 8 (21.33 ± 1.12). The average number of bees returning back over the eight weeks is shown in Figure 4.

The impact of four different artificial diets on the number of honeybees carrying pollen while entering the hive over an eight-week period was determined. All treatments showed a significant difference (F_(3, 158)_ = 1565.27, *p* < 0.001). T1 exhibited the highest average number of pollen-carrying bees (34.9 ± 0.46), followed by T3 (22.7 ± 0.35) and T2 (15.4 ± 0.33), with the lowest average number of pollen-carrying bees observed in T0 (4.10 ± 0.26).

**Figure 5:**
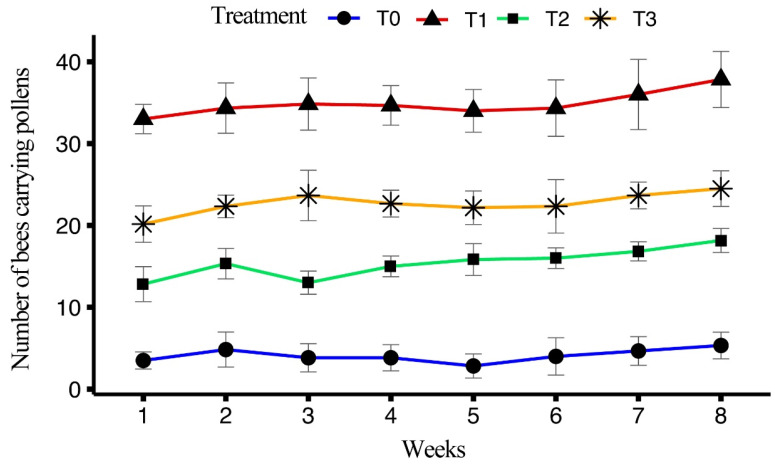
Effect of different artificial diets on foraging activity in terms of honeybees carrying pollen over a period of eight weeks.

In T1, the maximum number of pollen-carrying bees was recorded in week 8 (37.83 ± 1.40), with a steady increase observed from week 1 (33.00 ± 0.73) through the later weeks. For T2, the highest number of pollen-carrying bees was recorded in week 8 (18.17 ± 0.60), with week 7 and week 6 following closely, while the lowest number was observed in week 1 (12.83 ± 0.87). In T3, the maximum number of pollen-carrying bees was recorded in week 8 (24.50 ± 0.89), with weeks 7 and 6 also showing high numbers and the lowest was seen in week 1 (20.17 ± 0.91). For T0, the highest number of pollen-carrying bees was recorded in week 8 (5.33 ± 0.67), with weeks 7 and 6 following and the lowest was observed in week 5 (2.83 ± 0.60). The average number of pollen-carrying bees over the eight weeks is shown in Figure 5.

### 3.4. Honey Quality

The impact of four different artificial diets on honey quality was measured after an eight-week feeding period, and significant differences were observed among all the parameters. The pH of honey was highest in T1 (3.85 ± 0.03), followed by T3 (3.66 ± 0.02), T2 (3.51 ± 0.03) and the lowest in T0 (3.38 ± 0.03). Diastase activity, an indicator of enzyme content, was also highest in T1 (13.74 ± 0.19 units/g), followed by T3 (11.51 ± 0.12 units/g) and T2 (10.49 ± 0.18 units/g), with the lowest activity in T0 (6.24 ± 0.28 units/g). In terms of moisture content, T0 had the highest level (19.44 ± 0.28%), while T1 (16.91 ± 0.09%), T2 (16.32 ± 0.05%) and T3 (15.87 ± 0.12%) showed more moderate moisture levels. Ash content, a measure of mineral impurities, was lowest in T1 (0.17 ± 0.03%), moderate in T3 (0.36 ± 0.03%), and higher in T2 (0.55 ± 0.02%), with T0 having the highest ash content (0.95 ± 0.06%). Electrical conductivity was highest in T1 (0.94 ± 0.02 mS/cm), followed by T3 (0.76 ± 0.02 mS/cm), T2 (0.41 ± 0.01 mS/cm) and lowest in T0 (0.20 ± 0.02 mS/cm), which correlates with mineral presence. Mineral content was greatest in T1 (406.54 ± 4.10 mg/kg), followed by T3 (320.11 ± 5.08 mg/kg), T2 (225.68 ± 6.76 mg/kg), and lowest in T0 (199.83 ± 4.56 mg/kg) (Table 2). 

In terms of fructose content, T1 had the highest levels (396.21 ± 8.31 mg/kg), followed by T3 (352.33 ± 2.64 mg/kg), T2 (312.90 ± 4.66 mg/kg) and the lowest in T0 (307.45 ± 3.21 mg/kg). The total phenolic content, a measure of antioxidant properties, was also highest in T1 (60.50 ± 0.57 mg GAE/100 g), with moderate levels in T3 (28.51 ± 0.58 mg GAE/100 g), lower in T2 (15.40 ± 0.49 mg GAE/100 g) and the lowest in T0 (11.55 ± 0.44 mg GAE/100 g). Flavonoid content was similarly highest in T1 (44.41 ± 0.60 mg QE/100 g), followed by T3 (25.90 ± 0.88 mg QE/100 g), T2 (19.05 ± 0.66 mg QE/100 g) and lowest in T0 (4.72 ± 0.35 mg QE/100 g). Finally, the ORAC value, which reflects antioxidant capacity, was highest in T1 (10,237.30 ± 372.50 µmol TE/g), followed by T3 (7879.29 ± 253.01 µmol TE/g), T2 (6740.46 ± 234.01 µmol TE/g) and the lowest ORAC value was found in T0 (4998.71 ± 137.65 µmol TE/g). Overall, T1 demonstrated the best honey quality across nearly all parameters, while T0 consistently showed the lowest quality (Table 2).

### 3.5. Social Interactions

#### 3.5.1. Trophallaxis Events

The impact of four different diets on the number of trophallaxis events among honeybees over an eight-week period was analyzed. ANOVA results showed a significant effect of treatment on trophallaxis events (F_(3, 160)_ = 219.306, *p* < 0.001), while no significant differences were found for the week (F_(7, 160)_ = 0.941, *p* = 0.477) or the interaction between treatments and the week (F_(21, 160)_ = 0.393, *p* = 0.992).

**Figure 6:**
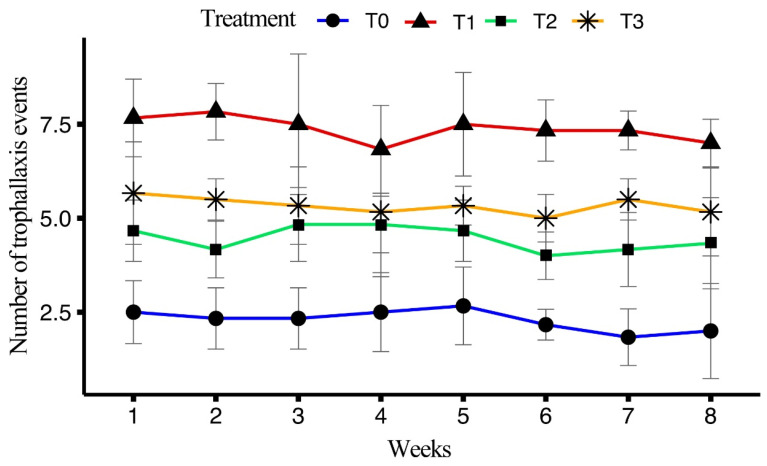
Effect of different artificial diets on trophallaxis events over the period of eight weeks.

T1 exhibited the highest average number of trophallaxis events (7.38 ± 0.15), followed by T3 (5.33 ± 0.141), T2 (4.46 ± 0.126), and T0 (2.29 ± 0.12). In T1, the highest number of trophallaxis events was recorded in week 1 (7.67 ± 0.42), with a gradual decrease observed, culminating in week 8 (7.00 ± 0.26). For T2, the maximum occurred in week 1 (4.67 ± 0.33), followed by a slight decline, ending with week 8 at 4.33 ± 0.49. In T3, the highest was observed in week 1 (5.67 ± 0.56), with week 8 showing a count of 5.17 ± 0.48. For T0, the maximum number of trophallaxis events was recorded in week 5 (2.67 ± 0.42), with the lowest in weeks 7 (1.83 ± 0.31) and 8 (2.00 ± 0.52). The average number of trophallaxis events over the eight weeks is clarified in Figure 6.

#### 3.5.2. Trophallaxis Time

The impact of four different diets on trophallaxis (seconds) over a period of eight weeks was analyzed. ANOVA results indicated a significant effect of treatment on the outcomes (F_(3, 128)_ = 166.423, *p* < 0.001), while no significant differences were found for the session (F_(15, 128)_ = 1.321, *p* = 0.199) or the interaction between the treatment and the session (F_(45, 128)_ = 0.604, *p* = 0.97).

**Figure 7:**
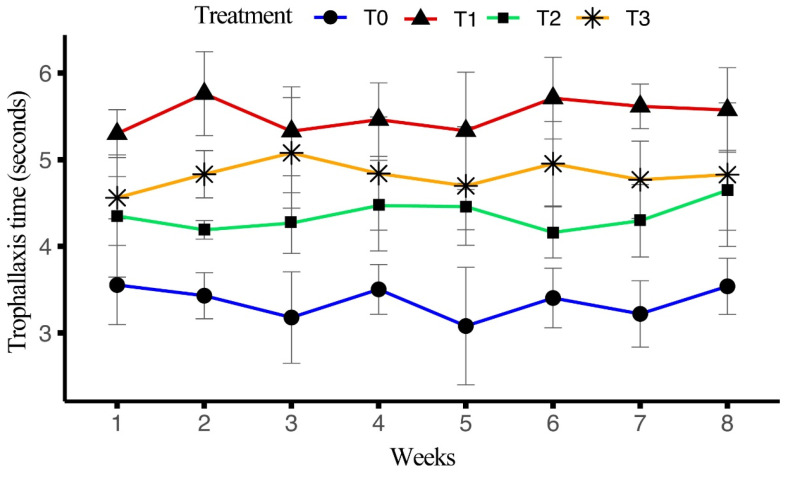
Effect of different artificial diets on trophallaxis time (seconds) over the period of eight weeks.

T1 exhibited the highest average trophallaxis time (5.51 ± 0.06 s), followed by T3 (4.82 ± 0.07 s), T2 (4.35 ± 0.06 s), and T0 (3.36 ± 0.0621 s). In T0, the highest trophallaxis time was recorded in week 1 (3.55 ± 0.19 s), with a gradual decline observed through week 5 (3.08 ± 0.28 s), before recovering slightly by week 8 (3.54 ± 0.13 s). For T1, the highest trophallaxis time was recorded in week 2 (5.76 ± 0.20 s), with consistent values throughout the remaining weeks, ending at 5.57 ± 0.20 s in week 8. In T2, the maximum occurred in week 1 (4.35 ± 0.29 s), with a slight decline towards week 8 (4.65 ± 0.19 s). For T3, the highest was observed in week 3 (5.08 ± 0.26 s), with values fluctuating slightly, finishing at 4.83 ± 0.34 s in week 8. The average trophallaxis times across the sessions are summarized in Figure 7.

#### 3.5.3. Trophallaxis Gathering in Each Event

The impact of four different diets on the number of bees in each trophallaxis event was analyzed. ANOVA results indicated a significant effect of treatment on the outcomes (F_(3, 128)_ = 141.173, *p* < 0.001), while no significant differences were found for the session (F_(15, 128)_ = 1.255, *p* = 0.241) or the interaction between the treatment and the session (F_(45, 128)_ = 0.609, *p* = 0.971).

**Figure 8:**
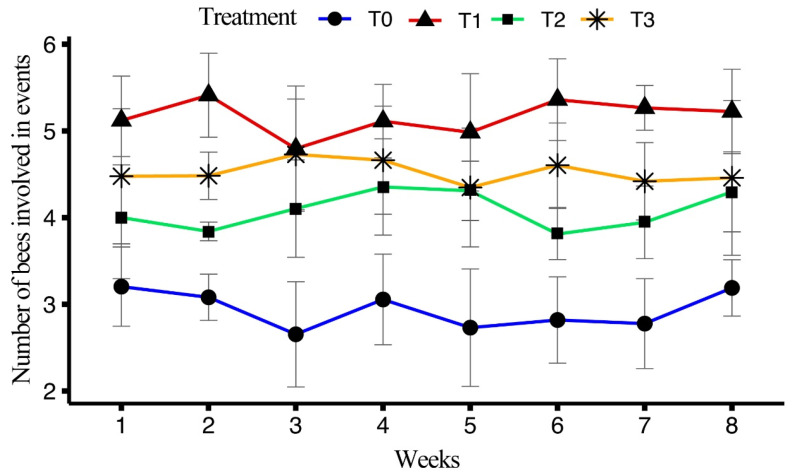
Effect of different artificial diets on number of bees in each trophallaxis event over a period of eight weeks.

T1 exhibited the highest average number of bees per trophallaxis event (5.16 ± 0.07), followed by T3 (4.52 ± 0.08), T2 (4.08 ± 0.06), and T0 (2.94 ± 0.07). For T1, the highest number of bees was recorded in week 2 (5.41 ± 0.20), with values remaining relatively consistent, ending at 5.22 ± 0.20 in week 8. In T2, the maximum occurred in week 1 (4.00 ± 0.29), with a slight decline towards week 8 (4.30 ± 0.19). For T3, the highest was observed in weeks 1 and 2 (4.48 ± 0.32 and 4.48 ± 0.11, respectively), with values fluctuating slightly, finishing at 4.46 ± 0.36 in week 8. In the control treatment, the highest number of bees was recorded in week 1 (3.20 ± 0.19), with a gradual decline observed through week 5 (2.73 ± 0.28), before recovering slightly by week 8 (3.19 ± 0.13). The number of bees in each trophallaxis event over the eight weeks is explained in Figure 8.

#### 3.5.4. Antennation Frequency

The impact of four different diets on antennation frequency was analyzed over a period of eight weeks. ANOVA results indicated a significant effect of treatment on antennation frequency (F_(3, 128)_ = 313.006, *p* < 0.001), while no significant differences were found for the session (F_(15, 128)_ = 0.869, *p* = 0.600) or the interaction between the treatment and the session (F_(45, 128)_ = 1.253, *p* = 0.166).

**Figure 9:**
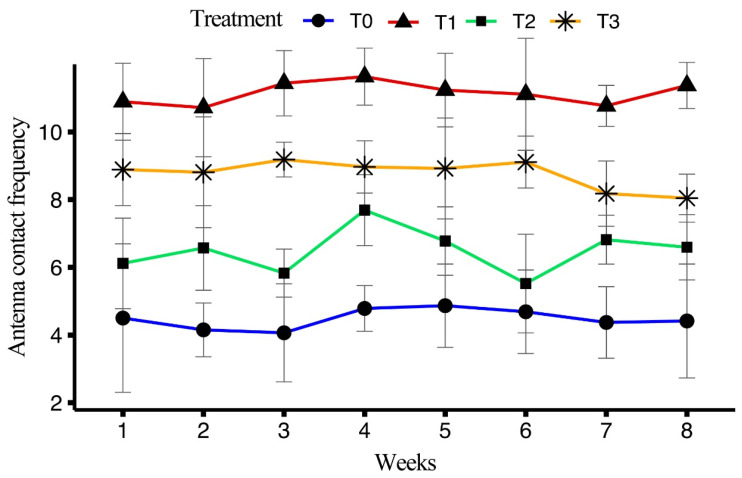
Effect of different artificial diets on antennation frequency over the period of eight weeks.

T1 exhibited the highest average antennation frequency (10.1 ± 0.13), followed by T3 (7.98 ± 0.13), T2 (5.96 ± 0.15), and T0 (4.18 ± 0.16). For T1, the highest frequency was recorded in week 3 (10.36 ± 0.35), with consistently high values maintained throughout, finishing at 10.30 ± 0.25 in week 8. In T2, the maximum occurred in week 2 (6.04 ± 0.45), with a gradual decline towards week 8 (6.05 ± 0.35). For T3, the highest was observed in week 1 (8.09 ± 0.39), with slight fluctuations throughout the weeks, finishing at 7.34 ± 0.26 in week 8. In T0, the highest antennation frequency was recorded in week 1 (4.20 ± 0.79), with values fluctuating slightly over the weeks, ending at 4.12 ± 0.61 in week 8. Antennation frequencies across the weeks are brief in Figure 9.

#### 3.5.5. Correlation Matrix for Social Interactions

The correlation matrix reveals strong positive relationships among four key parameters related to honeybee behavior: trophallaxis events, trophallaxis time in seconds, number of bees involved, and antenna contact frequency. Specifically, trophallaxis events show a strong correlation with trophallaxis time (0.7907), number of bees involved (0.7839), and antenna contact frequency (0.7964), indicating that increased food-sharing activities are linked to longer interaction durations and more participants. Trophallaxis time exhibits an even stronger correlation with the number of bees involved (0.9633), suggesting that longer feeding interactions are associated with greater participation. Additionally, a moderate to strong correlation exists between the number of bees involved and antenna contact frequency (0.7624), highlighting that as more bees engage in trophallaxis, the frequency of antennation also increases. These findings underscore the interconnectedness of these behaviors in enhancing communication and resource sharing within the bee colonies. The correlation matrix table is described below in Table 3.

## 4. Discussion

The study evaluated the effects of different artificial diets on the health biomarkers and performance metrics of *Apis mellifera* colonies during a dearth period, focusing on immune function, stress response, health biomarkers and foraging activity, honey quality, and social interactions in performance metrics. Significant differences in phenol-oxidase activity were observed across treatments, which is similar to the study of Sagona et al. [25], who noted that PO activity was directly influenced by the composition of the diet of honeybees. In our study, bees from T1 exhibited the highest PO activity (28.7 ± 1.20 U/mg of hemolymph), which suggests that it provided the ideal diet and most effectively supported the immune function of the bees. These findings were consistent with previous studies that emphasized the link between high-quality diets and enhanced PO activity [24]. T0 indicates a weak response (8.66 ± 0.81 U/mg of hemolymph), likely due to nutritional deficiencies and lower consumption of diet, supporting the hypothesis that poorer diets result in lower immune system function [24]. Diets T2 and T3 provided moderate nutritional support, resulting in less effective but still enhanced immune responses compared to T0 which also aligns with the findings of Wilson et al. [40] that the varying PO activity is linked to diet quality and to immune system robustness.

It had been studied in previous findings that, heat shock proteins (HSPs) such as Hsp90, Hsp70, Hsp82, Hsp60, Hsp40, and Hsp20 are expressed in *A. mellifera* due to different stress factors, including infections, pesticide exposure, and thermal stress [31,41,42]. These stress responses are crucial in helping honeybees to manage environmental pressures. In our findings, SDS-PAGE demonstrated that the expression of HSPs differs according the artificial diets offered to bees. The bees fed on the T1 diet exhibited Hsp70 and experienced the least stress which is consistent with previous studies by Chacon-Almeida et al. [41], who described that Hsp70 and Hsp82 are upregulated under heat stress. Lower expressions in T1 suggests that this diet helped mitigate the stress response. T0 exhibited the highest number of HSP bands, including Hsp40, Hsp60, and Hsp70, which aligns with the findings of Alqarni et al. [30], who reported that Hsp40, Hsp60, and Hsp70 are commonly expressed in response to stress conditions. The fewer HSP bands in T1 indicate that this diet provided better nutritional support, allowing bees to cope more effectively with heat stress. Diets T2 and T3 showed intermediate HSP expression, with both exhibiting Hsp60 and Hsp70. T2 displayed slightly more HSP bands than T3, suggesting that bees fed on T2 experienced somewhat higher stress levels than those on T3. This pattern reflects the partial protective effects of artificial diets in moderating the heat stress response.

In foraging experiments, the highest mean number of outgoing bees was observed in T1 (81.8 ± 1.37 bees) which indicates that the bees fed on T1 had a higher potential for foraging. Similar findings were observed by Delaplane et al. [43], Avni et al. [44] and Wright et al. [45] who reported that protein-enriched diets increased energy levels in bees. T1 showed the highest number of outgoing bees in week 1 (94.67 ± 2.86 bees), which gradually declined by week 8 (70.5 ± 3.45 bees). This trend suggests that while bees initially exhibit high energy levels, factors like diet composition and time may influence their foraging activity over the course of the experiment. Comparable trends were observed in T2 and T3, with T0 consistently exhibiting the lowest number of outgoing bees, indicating the limited efficacy of the control diet in supporting foraging behavior. A similar trend was observed in returning bees and pollen-carrying bees, supporting the notion that protein-rich diets enhance both foraging and pollen collection [43,45].

In the case of honey quality, a lower moisture level in the diets T1, T2, and T3 was observed with T1 showing the most desirable levels (16.91 ± 0.09%), which is consistent with previous studies [46,47]. On the other hand, T0 exhibited the highest moisture content (19.44 ± 0.28%), indicating higher potential for fermentation. Lower moisture levels reduce the possibility of fermentation by yeast and increase shelf life [14]. The pH of honey was found to be highest in T1 (3.85 ± 0.0318), which is in line with studies showing that honey with higher acidity is less prone to microbial growth [37]. Diastase activity was also recorded as being highest in T1 (13.74 ± 0.19 units/g). The findings highlight that honey produced under T1 conditions retained better enzymatic activity, which correlates with longer shelf stability [1]. Ash content, which reflects the mineral content of honey [14], was lowest in T1 (0.17 ± 0.03%) and highest in T0 (0.95 ± 0.06%) which aligns with the findings of Bogdanov et al. [36] that lower ash content is often associated with higher honey purity. High mineral contents were found in T1; our findings align with Terrab et al. [47], who described that high mineral content enhanced honey quality. Fructose was found in the highest concentration in T1 (396.21 ± 8.31 mg/kg), confirming previous studies by Aparna and Rajalakshmi [48] who described fructose as the dominant sugar in honey. The antioxidant activity, as measured by phenolic and flavonoid content, was also highest in T1. Phenolic content and flavonoid content indicate superior antioxidant properties in T1 honey. These results fall within the ranges reported by previous studies [37,49,50] that describe that the phenol and flavonoids enhance honey’s antioxidant capacity. The ORAC value was highest in T1 (10,237.30 ± 372.50 µmol TE/g), which correlates with the previous studies about the presence of phenolic compounds and flavonoids, supporting the claim that antioxidants in honey help protect cells from free radical damage [51].

In terms of social interactions, T1 exhibited the highest frequency of trophallaxis events (7.38 ± 0.15) and the longest trophallaxis time (5.51 ± 0.07 s), which suggests that the artificial diet T1 enhanced these social interactions. The results align with previous findings that improved nutrition causes an increase in social interactions, which are essential for efficient resource distribution and communication [39]. Similarly, T1 showed the highest number of bees involved per trophallaxis event and the highest antennation frequency indicated that a richer diet promotes greater social engagement [39]. T0 presented the lowest values for both parameters (bees/event and antennation frequency), indicating the poor social interactions due to the nutrition gap. Overall, the performance of T1 was the best, likely due to the combination of ingredients in the diet, which may enhance flavor and aroma, potentially increasing intake and providing synergistic effects on honeybee health and performance.

## 5. Conclusions

On the basis of the results, it was concluded that T1 (diet-1: Watermelon juice 20 mL + Fenugreek powder 2 g + Chickpea flour 20 g + Lupin flour 20 g + Mung bean flour 20 g + Yeast 10 g + powdered sugar 40 g + vegetable oil 10 mL) significantly improves the health and performance of *A. mellifera* colonies during dearth periods. T1 increased the immunity of bees, reduced stress, and enhanced foraging activity, honey quality and social interactions compared to the other tested diets. Nevertheless, T1 is a progressive and viable alternative for maintaining colony health and productivity under challenging environmental conditions when no flora is available for bees to feed on. Therefore, further research is needed to assess the long-term impacts of these diets on health and performance by evaluating other parameters across varying climatic zones, as environmental differences may affect the diet’s efficiency.

## Figures and Tables

**Table 1:** Heat Shock Protein (HSP) expression in honeybees at 45 °C.

Diet	Expression of HSPs	Protein Size (kDa)
T0	Hsp40, Hsp60, Hsp70	40, 60, 70
T1	Hsp70	70
T2	Hsp60, Hsp70	60, 70
T3	Hsp60, Hsp70	60, 70

**Table 2:** Describes the different artificial diets’ effects on quality parameters of honey (pH, Diastase activity, Moisture content, Ash content, Electrical conductivity, Minerals, Fructose, Total Phenolic content, Flavonoids, and ORAC) with respect to control diet group. The mean and standard error (SE) are provided along with the statistical classification indicated by letters (a, b, c, d) as per the significance.

Diets		pH	Diastase	Moisture	Ash Content	E.C	Minerals	Fructose	Phenol	Flavonoids	ORAC
		Mean ± SE	Mean ± SE	Mean ± SE	Mean ± SE	Mean ± SE	Mean ± SE	Mean ± SE	Mean ± SE	Mean ± SE	Mean ± SE
T1		3.85 ± 0.03 a	13.74 ± 0.19 a	16.91 ± 0.09 a	0.17 ± 0.03 d	0.94 ± 0.02 a	406.5 ± 4.10 a	396.21 ± 8.31 a	60.50 ± 0.57 a	44.41 ± 0.60 a	10,237 ± 372.50 a
T2		3.51 ± 0.03 c	10.49 ± 0.18 c	16.32 ± 0.05 c	0.55 ± 0.02 b	0.41 ± 0.01 c	225.7 ± 6.76 c	312.90 ± 4.66 c	15.40 ± 0.49 c	19.05 ± 0.66 c	6740 ± 234.01 c
T3		3.66 ± 0.02 b	11.51 ± 0.12 b	15.87 ± 0.12 b	0.36 ± 0.03 c	0.76 ± 0.02 b	320.1 ± 5.08 b	352.33 ± 2.64 b	28.51 ± 0.58 b	25.90 ± 0.88 b	7879 ± 253.01 b
T0		3.38 ± 0.03 d	6.24 ± 0.28 d	19.44 ± 0.28 d	0.95 ± 0.06 a	0.20 ± 0.02 d	199.8 ± 4.56 d	307.45 ± 3.21 d	11.55 ± 0.44 d	4.72 ± 0.35 d	4998 ± 137.65 d
ANOVA	d.f	3	3	3	3	3	3	3	3	3	3
Results	f	46	247	96.8	86.7	459.00	327.00	62.80	1806.00	640.00	69.80
	*p*	<0.001	<0.001	<0.001	<0.001	<0.001	<0.001	<0.001	<0.001	<0.001	<0.001

**Table 3:** Describes correlation among social interaction metrics in *A. mellifera*: trophallaxis events, trophallaxis time (seconds), number of bees involved in trophallaxis events and antenna contact frequency.

Variables	Trophallaxis Events	Trophallaxis Time	Number of Bees	Antennation Frequency
Trophallaxis Events	r = 1 (*p* < 0.001)	r = 0.7907 (*p* < 0.001)	r = 0.7839 (*p* < 0.001)	r = 0.7964 (*p* < 0.001)
Trophallaxis Time (s)	r = 0.7907 (*p* < 0.001)	r = 1 (*p* < 0.001)	r = 0.9633 (*p* < 0.001)	r = 0.7624 (*p* < 0.001)
Number of Bees Involved	r = 0.7839 (*p* < 0.001)	r = 0.9633 (*p* < 0.001)	r = 1 (*p* < 0.001)	r = 0.7379 (*p* < 0.001)
Antennation Frequency	r = 0.7964 (*p* < 0.001)	r = 0.7624 (*p* < 0.001)	r = 0.7379 (*p* < 0.001)	r = 1 (*p* < 0.001)

## Data Availability

Complete data are available in the manuscript. Further inquiries can be directed to the corresponding authors.
